# Targeted treatment for osteoarthritis: drugs and delivery system

**DOI:** 10.1080/10717544.2021.1971798

**Published:** 2021-09-13

**Authors:** Liwei Mao, Wei Wu, Miao Wang, Jianmin Guo, Hui Li, Shihua Zhang, Jiake Xu, Jun Zou

**Affiliations:** a School of Kinesiology, Shanghai University of Sport, Shanghai, China; b School of Biomedical Sciences, The University of Western Australia, Perth, Australia

**Keywords:** Osteoarthritis, intra-articular treatment, nanoparticles, drug delivery system, targeted treatment

## Abstract

The management of osteoarthritis (OA) is a clinical challenge due to the particular avascular, dense, and occluded tissue structure. Despite numerous clinical reports and animal studies, the pathogenesis and progression of OA are still not fully understood. On the basis of traditional drugs, a large number of new drugs have been continuously developed. Intra-articular (IA) administration for OA hastens the development of targeted drug delivery systems (DDS). OA drugs modification and the synthesis of bioadaptive carriers contribute to a qualitative leap in the efficacy of IA treatment. Nanoparticles (NPs) are demonstrated credible improvement of drug penetration and retention in OA. Targeted nanomaterial delivery systems show the prominent biocompatibility and drug loading-release ability. This article reviews different drugs and nanomaterial delivery systems for IA treatment of OA, in an attempt to resolve the inconsonance between *in vitro* and *in vivo* release, and explore more interactions between drugs and nanocarriers, so as to open up new horizons for the treatment of OA.

## 1. Introduction

**Figure 1.**
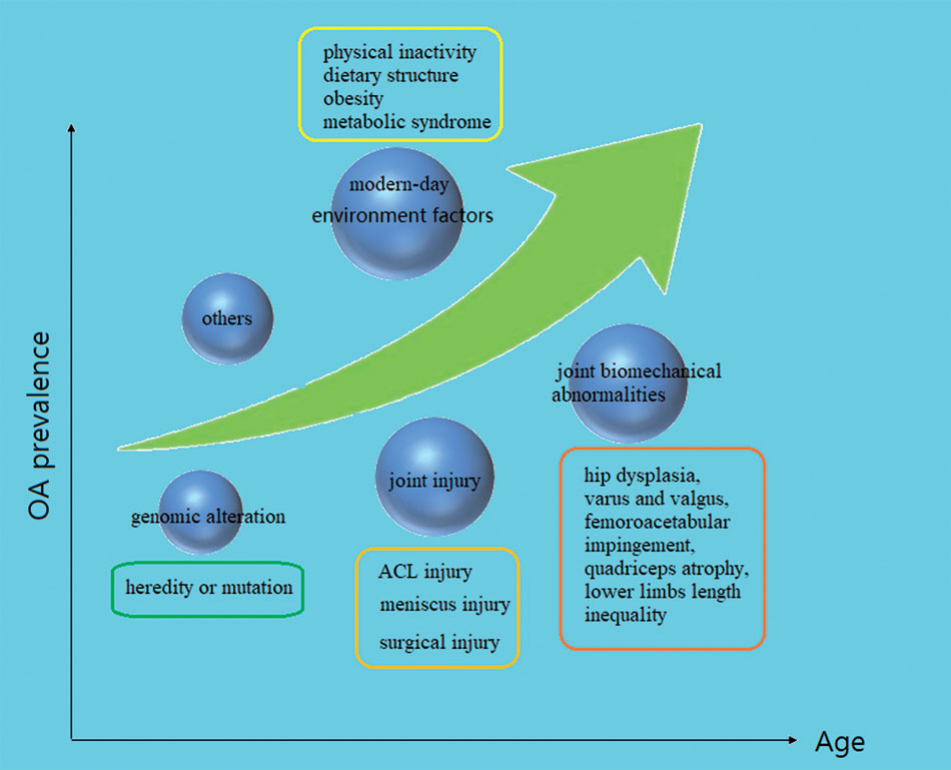
Risk factors of OA prevalence alone with the rising of age.

As life expectancy has increased over the past half-century, the prevalence of Osteoarthritis (OA) has been increasing prominently. In Europe, the United States and other developed countries, 10%−15% of adults over 60 years old suffer from OA due to the severity of the aging population, with a significantly higher prevalence rate in older women than older men (Bijlsma et al., 2011; Prieto-Alhambra et al., 2014; Wallace et al., 2017). Substantial evidence indicates that age, as the strongest risk factor for OA, interacts with multiple risk factors throughout the pathological process. Obesity is another non-negligible risk factor for OA, increasing the risk by more than three times (Blagojevic et al., 2010; Silverwood et al., 2015; Reyes et al., 2016). Obesity increases the load on the large joints of lower limbs (hip, knee and ankle) and further affects the biomechanics of the joints. Meanwhile, the increase of adipokines and inflammatory cytokines caused by obesity promotes the development of OA (Kulkarni et al., 2016; Wang & He, 2018; Liu et al., 2019; Misra et al., 2019). Different types of joint injury are an important basis for the pathogenesis of OA. Long-term high-intensity exercise training is one of the susceptibility factors of OA and post-traumatic osteoarthritis (PTOA) is a representative type (Bodkin et al., 2020; Rothrauff et al., 2020). Injuries of the anterior cruciate ligament (ACL) and meniscus are the most common causes of PTOA (Ajuied et al., 2014; Li et al., 2019; Wang et al., 2020). Knee ligament, meniscus, muscle, bone, and tendon injuries or surgery increase the risk of knee arthritis by at least four times (Muthuri et al., 2011; Poulsen et al., 2019). The joint biomechanical abnormalities caused by congenital or acquired joint anatomical dysfunction may lead to the occurrence of OA under the influence of self and environmental factors. Hip dysplasia, varus and valgus, femoroacetabular impingement, quadriceps atrophy, lower limbs length inequality will affect the pathological process of OA in varying degrees (Nishida et al., 2017; Wyles et al., 2017; Kim et al., 2018; Lynch et al., 2019; Hernandez et al., 2020; Springer et al., 2020; Xu et al., 2020). Genomic studies of OA patients and their families have revealed new biogenetics insights of OA pathogenesis and multiple gene loci were found relevant to the pathogenesis of OA (Zeggini et al., 2012). Similar to aging, changes in modern lifestyle also affect the incidence of OA. Physical inactivity and changes in dietary structure contribute to obesity and metabolic syndrome which result in abnormal regulation of bone metabolic factors such as dyslipidemia, impaired glucose tolerance and hypertension (Berenbaum et al., 2018) (Figure 1).

## 2. Pathogenesis of OA

**Figure 2.**
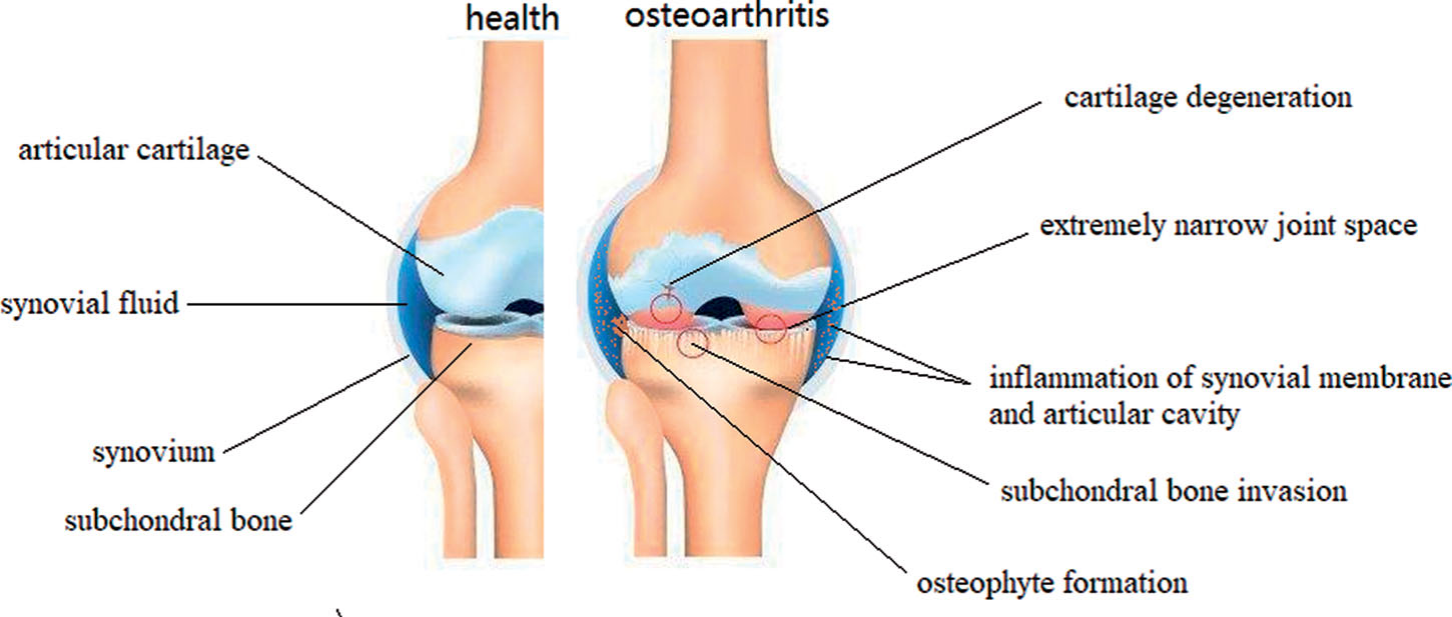
Schematic representation of healthy knee joint structure and pathological changes of knee osteoarthritis.

OA is the most common degenerative disease of the whole joint, progressively affecting the articular cartilage, synovium, subchondral bone, and periarticular tissues like ligaments, capsule, and periarticular muscles (Glyn-Jones et al., 2015; Martel-Pelletier et al., 2016; Sharma, 2021). The main pathological manifestations are degeneration of articular cartilage, thinning of subchondral bone, osteophyte formation around the joint, meniscal alterations, synovial fluid inflammation, ligament injury, and joint capsule hypertrophy (Hügle & Geurts, 2017; Roseti et al., 2019). Cardinal symptoms include pain, swelling or even deformity of the joints, stiffness (especially severe and transient morning stiffness), popping or crepitus during joint motion, and mobility disorder (Fu et al., 2018; Bacon et al., 2020; He et al., 2020). Traditionally, OA is regarded as a passive degenerative disease or injury caused by long-term wear and tear. However, new insights suggest that OA is actually an active dynamic process arising from imbalance of joint damage and repair. Initially, erosion begins on the surface of the cartilage and gradually deepens into the calcified cartilage area. During this process, chondrocytes attempt to repair the damage by enhancing proliferation and differentiation, but the accompanying inflammatory response inhibits chondrocyte function. Then the subchondral bone proliferates pathologically and erodes the cartilage layer. The endochondral pathologic enhancement of osteogenesis results in the formation of osteophytes around the joint margins (Figure 2).

## 3. Management of OA

### 3.1. Non-pharmaceutical strategies

Almost all guidelines recommend regular and individualized exercise in the different pathological stages of OA. The most common exercise for OA treatment includes aquatic exercise, aerobics, resistant exercises, multimodal and combined exercise (Luan et al., 2019). However, the effect of exercise intensity on the outcome of OA rehabilitation has not been fully elucidated, especially in the acute stage of OA. Inappropriate exercise prescription may aggravate the development of the disease.

Physical therapy (PT) has a prominent therapeutic effect on OA including therapeutic ultrasound, electrical stimulation, phototherapy, hydrotherapy, magnet therapy, cryotherapy and thermotherapy. PT has a significant relief effect on the symptoms of osteoarthritis, including pain, edema, and joint motion disorders, which is suitable for emergency management in the acute phase (de Oliveira Melo et al., 2016; Aciksoz et al., 2017; Rothenberg et al., 2017; Langella et al., 2018). Physical factors are also effective triggers for stimuli-responsive NPs for controllably releasing agents in IA treatment and this will be introduced blew.

OA patients usually require assistive devices to compensate for decreased strength, impaired balance, pain during movement. Common devices include splints, braces, walking canes, functional footwear and other training equipment. Splint shows remarkable improvement of pain relief for base-of-thumb osteoarthritis with time dependence of efficacy (Rannou et al., 2009; Gomes Carreira et al., 2010; Becker et al., 2013). Daily cane use can diminish pain and maintain a normal gait which is crucial for OA patients to preserve joint function and muscle strength in the early stage of rehabilitation (Jones et al., 2012; Moe et al., 2012). Although clinical studies have yielded some positive results, questions remain about the necessity for assistive devices and their long-term safety.

**Figure 3.**
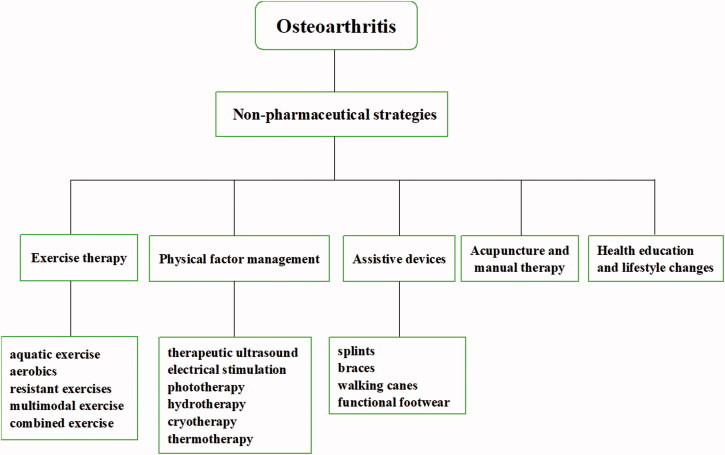
Non-pharmaceutical strategies of OA.

Acupuncture is a non-pharmaceutical treatment of traditional Chinese medicine (TCM). Acupuncture plays a certain role in relieving pain and restoring function for OA treatment. The therapeutic effect of acupuncture may come from the regulation of inflammatory factors (Lin et al., 2020; Shi et al., 2020). However, evidence showed incertitude of acupuncture in treating OA, in particular the obvious difference between electroacupuncture and manual acupuncture (Wang et al., 2020; Tu et al., 2021), and in a small sample study, no difference was observed in the eight-week (three sessions per week) acupuncture intervention (Lin et al., 2018). In addition, non-pharmaceutical strategies include health education, lifestyle changes such as diet, physical activity, and weight control, and Self-management is an important measure to prevent OA (Figure 3).

### 3.2. Pharmacological management

Considering that many patients with OA are unable to identify independent risk factors for intervention and there is still uncertainty about the efficacy and adaptability of non-pharmacological treatment. Pharmaceutical drugs remain the primary treatment for OA including topical, oral and injectable intervention. First-line drugs include non-steroidal anti-inflammatory drugs (NSAIDs), paracetamol, analgesics, capsaicin, and glucocorticoids and show remarkable efficacy in symptom control (Hochberg et al., 2012; Bannuru et al., 2019; Kolasinski et al., 2020). However, both topical and oral drug use have certain limitations in that topical drugs have lower systemic drug levels than oral, but they are limited by drug penetration, retention and high-frequency medication. In addition, long-term use of NSAIDs and cyclooxygenase 2 (COX2) is an inconvenient risk of gastrointestinal and cardiovascular systems (Nissen et al., 2016; Chan et al., 2017; Solomon et al., 2017). With the deepening of research, an increasing number of drugs are being developed for the treatment of OA.

Another class of commonly used drugs is referred to as cartilage protectors including glucosamine and chondroitin sulfate products. Although some studies have shown the anti-inflammatory and analgesic effects of chondroitin and glucosamine in OA treatment, thus alleviating clinical symptoms and delaying disease progression, the effect is only better than that of a placebo, lacking favorable evidence and relevancy (Bruyère et al., 2014; Sharma, 2021). Hyaluronic acid is a high-molecular-weight GAG in synovial fluid and cartilage which is widely used as a viscous supplement to lubricate joints and absorb impact. A large number of meta-analysis results showed that the clinical efficacy and outcome correlation of hyaluronic acid was not explicit, and the new treatment guidelines no longer recommend hyaluronic acid for OA treatment (Rutjes et al., 2012; McAlindon et al., 2014).

### 3.3. Surgical management

Surgical treatment is suitable for severe OA caused by some specific etiological factors like trauma, congenital joint deformity and dysplasia, osteonecrosis. Common techniques include arthroscopic debridement and lavage, cartilage transplantation, meniscus resection and arthroplasty. Arthroscopic debridement shows obvious clinical results in elbow OA, and joint foreign body and impingement are considered potential indications (MacLean et al., 2013; Carlier et al., 2019; French Arthroscopic Society, 2019). Some studies showed that arthroscopic debridement and lavage are also effective in the improvement of symptoms in shoulder, knee and thumb OA (Furia, 2010; Bexkens et al., 2018; Lo Presti et al., 2020). However, some clinical trials and the systematic review showed unsatisfactory results for debridement and lavage procedures that the outcomes after surgery are temporary or no different from placebo procedures (Moseley et al., 2002; Kirkley et al., 2008; Skelley et al., 2015). Although some minimally invasive procedures are well established, surgical treatment is also different degrees of trauma to the joint.

Osteochondral allograft transplantation (OCA) shows dependable improvement of pain, function and symptom scores in chondral lesions. In multiple follow-up studies over 10 to 20 years, OCA shows significant prognostic effects, greatly delayed the time of arthroplasty, and reduced reoperation rate (Frank et al., 2017; Pascual-Garrido et al., 2017; Stone et al., 2017; Ekman et al., 2018; Frank et al., 2018). Nonetheless, OCA shows definitely cost-effective which means the clinical outcomes are largely dependent on the overall cost of the operation, particularly the cost of the graft, which will increase the financial pressure on the patients (Mistry et al., 2019). OCA can help athletes return to competition, but there is a high probability of reoperation, requiring debridement and removal of the free body (Crawford et al., 2019). In addition, the study found an intractable relationship between the thickness of the graft and the prognosis that thin grafts may result in high risk of subchondral cysts and thicker grafts may delay osseous graft integration after surgery (Ackermann et al., 2019).

## 4. Targeted nanomaterial drug delivery systems

### 4.1. DDS targeting the inflammation of synovium and cartilage

Lornoxicam (Lnx) is a thienothiazine derivative NSAIDs of oxicam class with properties of anti-inflammatory, analgesic and joint repair (Berry et al., 1992; Kidd & Frenzel, 1996; Hall et al., 2009). However, due to poor aqueous solubility and upper digestive tract absorption, oral administration and simple interarticular injection have many restrictions, such as gastrointestinal reactions, low absorption rate, and rapid clearance rate (Hamza & Aburahma, 2009). Zhang (Zhang & Huang, 2012) et al evaluated the biocompatibility, systemic toxicity, retention time and anti-inflammatory effect of Lnx-loaded PLGA microspheres (Lnx-MS) in papain-induced OA rats. Lnx-MS showed remarkable retention time in joint tissue and persistent low level in plasma during 96 hours after injection. Lnx suspensions distribution in plasma peaked in 24 hours and they were cleared within 24 hours in joint tissue. Lnx-MS was verified to be biodegradable and safe to proliferate and differentiate chondrocytes. Pharmacodynamics showed a reduction in joint swelling and proteoglycan loss. Researchers used chitosan/tripolyphosphate MS carrying Lnx to intervene monosodium iodoacetic acid (MIA) induced OA rats. Compared to suspensions, chitosan MS prolonged drug delivery time to 8 days with a long-lasting anti-inflammatory effect (Abd-Allah et al., 2016). The optimized formula showed remarkable improvement in joint tissues and inflammatory cytokine inhibition.

Meloxicam (Mlx) is also a hydrophobic and lipophilic NSAID. Compared to other NSAIDs, Mlx is a preferential inhibitor of COX2 thus resulting in slight gastrointestinal reactions, but poor aqueous solubility limits the absorption and bioavailability. Shohreh Fattahpour (Fattahpour et al., 2020) et al compared two Mlx delivery systems that carboxymethyl chitosan-methylcellulose-pluronic hydrogel (CMC-MC-P hydrogels) containing Mlx NPs showed prominent biocompatibility, low degradation and swelling in comparison to hydrogel containing Mlx solution. The drug release studies showed that 85%-97% of Mlx in NPs released in 37 days while hydrogel-containing solution release almost 100% after 20 days. Chondrocytes have better proliferation and adhesion in NPs system and they are affected by the concentration of NPs and Mlx solution.

Celecoxib (Clx) is a kind of NSAID working as a COX-2 selective inhibitor and has significant anti-inflammatory and analgesic effects for OA (Davies et al., 2000; Hochberg et al., 2016; Puljak et al., 2017). Compared to nonselective NSAIDs, Clx shows certain drug safety in gastrointestinal symptoms and renal adverse events at an appropriate dose (Chan et al., 2010; Solomon et al., 2018; Yeomans et al., 2018). However, long-term use of aspirin can attenuate the gastrointestinal safety of Clx and cardiovascular safety is also a potential risk for oral administration of Clx (Silverstein et al., 2000; Nissen et al., 2016; Reed et al., 2018). Audrey Petit (Petit et al., 2014) et al evaluated the biocompatibility of Clx-loaded hydrogel carried on an acetyl-capped PCLA-PEG-PCLA triblock copolymer *in vitro* and *in vivo*. The copolymer is detected thermoset that it stays in a sol state at room temperature and turns into immobile gels at 37 °C. The study showed sustained topical Clx release both *in vitro* and *in vivo* (90 days and 4-8 weeks) based on polymer dissolution. No damage was observed in articular cartilage of healthy rats after subcutaneous injection of encapsulated Clx polymer thus revealing this Clx-loaded polymer as a safe DDS for OA treatment. Another formulation used endcapped PCLA-PEG-PCLA copolymer loaded with Clx showed similar results with acetyl-capped copolymer but longer release time (van Midwoud et al., 2018). These results suggest the potential of PCLA-PEG-PCLA for OA targeted injection treatment.

Polyesteramide (PEA) MS is another high-profile Clx-loaded NPs. Maarten Janssen (Janssen et al., 2016) et al reported that Clx was released sustainedly up to 80 days *in vitro* and inflammation responsive release was observed in the Hl-60 cell line. In OA-induced (ACLT + pMMx) rats, degradation of PEA MS was higher than health rats which verified inflammation responsive release again. Regrettably, no difference was observed in cartilage degeneration changes between 0.9% NaCl, single MS and Clx-loaded MS. A later similar study showed that Clx-loaded PEA MS reduced the osteophytes, subchondral ossifying, bone cysts, and synovial inflammation in surgery-induced OA rats (Tellegen et al., 2018). Ian J Villamagna (Villamagna et al., 2019) et al optimized the structure of PEA MS and demonstrated different toxicity *in vitro* and *in vivo*.

Recently, poly (D, L-lactic acid) (PDLLA) microparticles (MP) and hyaluronan nanocapsules were developed to load Clx for rats OA model research. Two different kinds of PDLLA MP (drug in solution MP and nano-drug embedded MP) showed good biocompatibility, drug loadings rate, entrapment efficiencies and long action sustained release of Clx. PGE2 decreased in IL-1β induced human articular synoviocytes after two MP interventions (Salgado et al., 2020). Clx-loaded hyaluronan nanocapsules showed remarkable entrapment efficiency and release time *in vitro*. In addition, nanocapsules improved knee joint swelling, morphology, histomorphology and inflammation in MIA-induced rats OA model (El-Gogary et al., 2020).

Diclofenac (Dcf) is also commonly used in the targeted treatment of OA by loading nanomaterials. Bryan B Hsu (Hsu et al., 2014) et al first reported a new polymer–Dcf conjugate system of biodegradable thin films using a layer-by-layer (LBL) self-assembly process and achieved sustaining small molecule release. Dcf is firstly activated with triethylene glycol (TriEG) to form a TriEG-Dcf prodrug conjugate and then conjugated to poly (l-glutamic acid) to complete integral PGA-TriEG-Dcf formulation assembly. After conjugating to poly(l-lysine) to form PLL/PGA-TriEG-Dcf, the release time increased significantly, exceeding several months. PLL/PGA-TriEG- Dcf showed a prominent anti-inflammatory effect by COX inhibition and no deleterious effects appeared in synthetic procedures. Adrian Sulistio et al. (Sulistio et al., 2017) developed a new polymer‐Dcf conjugate (PDCs) and found that PDCs provide high drug loading and a sustained steady release of Dcf. Notably, by regulating the feed ratio of PEG co‐monomers and the amount of PDCs, Dcf loading and release kinetics can be continuously optimized to achieve precise control. The hydrogel films of poly(n-vinylcaprolactam) NPs (νPVCL) were reported temperature-responsive on the basis of high drug loading by the LBL assembly technique (Zavgorodnya et al., 2017). After being loaded with Dcf, different layers of νPVCL show different performance of drug loading and release, and when (νPVCL)_30_ is in the artificial skin film at 30 °C (average skin temperature), the cumulative drug release in 24 hours is 12 times that of 22 °C. Kuan Zhang (Zhang et al., 2020) et al reported a dual-functional nanospheres PNIPAM-PMPC loaded with Dcf prepared by emulsion polymerization. PNIPAM-PMPC nanospheres show higher drug release at 37 °C than 22 °C. Meanwhile, it has prominent lubrication by forming a compact hydration layer outside. Dcf-loaded PNIPAM-PMPC nanospheres have good biocompatibility, which increase anabolic genes and inhibit catabolic genes of chondrocytes. Toshio Kawanami (Kawanami et al., 2020) et al developed a novel Dcf-hydrogel conjugate system produced by 2-pyridylamino-substituted 1-phenylethanol (PAPE) which reduced the production of the lactam in regular ester conjugates of Dcf. Besides, this hydrogel conjugate has an optimizable release rate regulated by physiological microenvironment. Recently, inartificial clay mineral attapulgite (ATP) was used to produce an enhanced supramolecular hydrogel by cyclodextrin pseudopolyrotaxane (PPR) system (Ha et al., 2021). This ATP hybrid hydrogel appears to sustained release of Dcf, good biocompatibility and remarkable anti-inflammatory *in vivo* test.

Etoricoxib (Ecx) is a highly hydrophobic selective inhibitor of COX-2 and is commonly used for acute pain caused by rheumatoid arthritis and OA. However, due to low solubility, severe pH dependence, and cardiovascular risk, oral or systemically administration of Ecx remains many challenges (Okumu et al., 2009). Polycaprolactone (PCL) MP is early used for Ecx-loaded targeted delivery. PCL MP showed satisfactory biocompatibility, hypotoxicity and long-term sustained release *in vivo* and *in vitro* tests (Arunkumar et al., 2016a). Loading PCL MP with chitosan gel to form novel injectable gel MP can enhance the duration of Ecx in synovial (Arunkumar et al., 2016b). Pingju Liu (Liu et al., 2019) et al developed novel PLGA-PEG-PLGA copolymer NPs loaded with Ecx. NPs showed sustained release *in vitro* and significant anti-inflammatory effects in subchondral bone, synovium, and cartilage *in vivo*. Alaa H Salama (Salama et al., 2020) et al reported Ecx-loaded PLA-CS NPs synthesized from polylactic acid and chitosan hydrochloride. By adjusting the ratio of surfactant, the formula with the smallest particle size and the most obvious slow-release effect was optimized. PLA-CS NPs showed cytocompatibility and enhanced ALP activity *in vitro* test.

In addition to chemically synthesized nanomaterials, organometallic materials are also used for drug-loaded targeted therapy. A UiO-66 metal-organic framework (MOF) was used as DDS for ketoprofen by introducing functional groups (NH_2_, NO_2_) (Li et al., 2019). Ketoprofen-loaded UiO-66-NH_2_ showed good biosafety and sustained drug release. NSAIDs are relatively mature in delivery systems research (Table 1).

**Table 1.** NSAIDs and delivery system in OA treatment.

Drugs	Delivery system	Physical properties	*In vitro* findings	Rat model	*In vivo* performance	Refers
Lnx	PLGA MS	None	None	4% papain induce	Low circulating concentration; long drug retention; biocompatibility safe; reduce joint swelling; histological improvement	Zhang & Huang (2012)
	Chitosan/TPP MS	Particle size (L9): 5.4 µm; EE%: 59.5%	pH and particle size dependent; Sustained release over 8days	3mg/50µl MIA induce	Prolongation of retention time; reduce joint swelling; inhibit IL6; histological improvement	Abd-Allah et al. (2016)
Mlx	CMC-MC-P hydrogels NPs	EE% (N2): 87.5%; Low degradation and swelling behavior	Sustained release: 85-97% after 37days; Promote proliferation and adhesion of chondrocytes	None	None	Fattahpour et al. (2020)
Clx	Acetyl-capped PCLA-PEG-PCLA thermogels	Thermosensitive gel (37 °C)	Sustained release in 90 days	Healthy	Sustained release 4–8 weeks; biocompatibility safe	Petit et al. (2014)
	PEA MS	Particle size: 10–100 µm	More degradation of PEA in inflammatory environment; Sustained release: over 80days	ACLT and pMMx surgery	Biocompatibility safe; sustained release over 12 weeks; reduce degradation of PEA; no cartilage pathology difference	Janssen et al. (2016)
	PEA MS	Average molecular weight: 70 kDa	Sustained release over 28 days	ACLT and pMMx surgery	Dose-dependently release over 120 hours; subchondral bone protection, osteophyte reduction, histological improvement	Tellegen et al. (2018)
	PDLLA MP	20 to 40 μm mean size; 10% to 50% w/w drug loading; EE%> 80%	Sustained release over 90 days; inhibit PGE2 release	None	None	Salgado et al. (2020)
Dcf	PLL/PGA-TriEG	Dfc loading densities: 295 μg/mm3 (30wt%) (40 bilayers); Thickness: 2.7 ± 0.5 μm	Sustained release over 14 months; inhibition of COX-1	None	None	Hsu et al. (2014)
	PEG-ELDI-PHB‐MG co‐monomer	Dfc loading: 38 w/w%	Sustained release 50–120 days	None	None	Sulistio et al. (2017)
	νPVCL nanogel	pH-dependent nanogel size; Temperature sensitive swelling	Temperature-responsive release; sustained release over 24 h at 32 °C	None	None	Zavgorodnya et al. (2017)
	PNIPAM-PMPC nanospheres	Average diameter: 237 ± 97.9; Zeta potential: −16.7 mV; EE%: 41.7%; LC%: 11.1%	Temperature-responsive: 66.8% and 73.4% release with 24 h and 72 h, 81.2% and 87.5% at 37 °C; lubrication; biocompatible, no cytotoxicity and chondroprotective	None	None	Zhang et al. (2020)
	ATP Hybrid-PPR Hydrogel	EE%: 77.3% to 96.4%	Good biocompatibility Sustained release 70%–90% over 4 days	100 µL of carrageenan solution (1%) induced	Prolong retention time to 7 days; Histological improvement	Ha et al. (2021)
Ecx	PCL MP; PCL-CICG MP	Average size: 16.26 ± 10.14 μm; EE%: 83.7 ± 3.27%; LC%: 2.67 ± 0.11%	Controlled release for 28 days; Bio-safe	Healthy	Prolong the retention time to 4 weeks in the synovia	Arunkumar et al. (2016)
	PLGA-PEG-PLGA NPs	Average diameter: 339 nm; Zeta potential: 1.68 ± 0.85 mV	Sustained release over 30 days; Bio-safe	ACLT surgery	Maintain ECM; histological improvement; subchondral bone protection	Liu et al. (2019)
	PLA- chitosan NPs	Smallest size: 420.30 ± 40.16 nm; Zeta potential: 25.95 ± 1.34mv; EE%: 92.15 ± 0.78%	Quick release after first 2 h, sustained release over 28 days; RE%: 55.02%; enhancing the proliferation of MC3T3-E1 cells; enhancing ALP activity and calcium ion concentration	None	None	Salama et al. (2020)
Kpf	UiO-66 metal-organic framework	Particle size: 30-50nm; Loading amount: 38w%	Quick release at the beginning 12 h, slow release in next 60 h; RE%: 65w%; Bio-safe	None	None	Li et al. (2019)

Abbreviations: Lnx: Lornoxicam; MS: microspheres; MP: microparticles; PLGA: Poly (lactic-co-glycolic acid); TPP: tripolyphosphate; EE%: entrapment efficiency%; MIA: monosodium iodoacetate; Mlx: Meloxicam; CMC-MC-P: carboxymethyl chitosan-methylcellulose-pluronic; Clx: Celecoxib; PCLA: poly(ε-caprolactone-co-lactide); PCLA-PEG-PCLA: poly(ε-caprolactone-co-lactide)-b-poly(ethylene glycol)-b-poly(ε-caprolactone-co-lactide); PEA: polyester amide; ACLT: anterior cruciate ligament transection; pMMx: partial medial meniscectomy; PDLLA: poly D,L-Lactic Acid; Dcf: Diclofenac; PLL: poly(l-lysine); PGA: poly (l-glutamic acid); TriEG: triethylene glycol; PHB: p-hydroxybenzoate; ELDI: ethyl‐L‐lysine diisocyanate; MG: monoglyceride; PVCL: poly(N-vinylcaprolactam); PNIPAM-PMPC: poly[N-isopropylacrylamide-2-methacryloyloxyethyl phosphorylcholine]; LC: loading capacity; ATP: attapulgite; PPR: pseudopolyrotaxane; Ecx: Etoricoxib; PCL: Poly caprolactone; CICGs: composite injectable Chitosan gel; ECM: extracellular matrix; RE: release efficiency.

### 4.2. DDS targeting cartilage protection and regeneration

Diacerein (Dcn) is a chondroprotective agent metabolized by acetyl esterases and it exerts anti-inflammatory and cartilage protective effects by metabolizing rhein (Jain et al., 2015; Lohberger et al., 2019). Dcn does not affect the production of prostaglandins, and there are few reports of gastrointestinal disorders, so it is recommended as a first-line drug for OA, especially for patients contraindicated to NSAIDs (Bartels et al., 2010; Pavelka et al., 2016). However, low water-solubility limits the oral bioavailability of both diazepine and rhein.

Achint Jain (Jain et al., 2013) et al developed Dcn-loaded solid lipid NPs (Dcn-SLN) by ultrasonication technique and characterized its physicochemical properties. Dcn-SLN shows sustained drug release *in vitro* and high bioavailability of oral management in a rat model. Mubashar Rehman (Rehman et al., 2015) et al optimized Dcn-SLN by mixing proportionally of solid and liquid lipids to form binary SLNs. This novel formula demonstrates not only sustained Dcn release but, more importantly, rapid release at high temperatures. This thermoresponsive release property makes it possible to combine it with OA thermal therapy. In subsequent studies, Dcn-loaded niosomes and self-nano emulsifying gel based on GLC and TPGS were successively developed (El-Say et al., 2016; Eltobshi et al., 2018). Both DDS showed good drug release performance *in vitro* and anti-inflammatory effect *in vivo* after optimization. Dcn or rhein-loaded PLGA NPs showed excellent biocompatibility and inflammatory inhibition *in vitro* (Gómez-Gaete et al., 2017; Jung et al., 2020). Dcn-loaded PLGA NPs could effectively protect the cartilage injury and inhibit the progression of inflammation after interarticular injection *in vivo*. Diana E Aziz (Aziz et al., 2018) et al developed a novel Dcn delivery system using elastosomes for transdermal delivery thereby avoiding oral adverse effects (Table 2).

**Table 2.** Cartilage protector and delivery system in OA treatment.

Drugs	Delivery system	Physical properties	*In vitro* findings	Rat model	*In vivo* performance	Refers
Dcn	SLN	Particle size: 382 ± 16 nm; Zeta potential: −1966 ± 21 mv TDC (%): 985 ± 02%	Sustained-release up to 12 h	Healthy	Anti-diarrhoeal; enhanced oral bioavailability;	Jain et al. (2013)
	SLNs-GNPs	Particle size: 6.9–9.7 nm; EE%: 79–97%; Zeta potential: −22.7– −38.6 mv	Thermoresponsive release; Sustained-release up to 72 h	None	None	Rehman et al. (2015)
	Glc-TPGS self-nanoemulsifying DDS	Uniform and homogeneous surface; Crystalline anhydrous nature; Drug stability	Enhanced drug release	0.1 mL of 1% w:v λ-carrageenan	Edema and inflammation inhibition; histopathological improvement; TNF-α and caspase-3 inhibition	Eltobshi et al. (2018)
	Niosomal gel- 3% HPMC	Particle size: 7.33- 23.72µm; EE%: 9.52%- 58.43%	Sustained-release up to 8 h	0.1 ml of 1% carrageenan	Edema and inflammation inhibition	El-Say et al. (2016)
	PLGA NPs	Particle sizes: 200– 320 nm; Loading efficiency: 81.76 ± 3.26% DIA (1%) and 80.85 ± 7.51% DIA (5%)	Sustained release up to 63 days; non-toxic; Inhibit IL-1, IL-6, TNF-α, MMP-3, MMP-13, COX-2, and ADAMTS-5	50 μL of 10 mg/mL MIA	Histological improvement; cartilage protection; inhibit inflammation	Jung et al. (2020)
	EA-based vesicular nanocarriers	EE%: 96.25 ± 2.19%; Particle sizes: 506.35 ± 44.61 nm; Zeta potential: −38.65 ± 0.91 mV	Sustained-release up to 8 h;	Transdermal delivery	Biocompatibility safe;	Aziz et al. (2018)
Rhein	PLGA MP	Mean diameter: 4.23 ± 0.87 μm; EE%: 63.8 ± 3.0%; Loading efficiency: 1.60 ± 0.07%; Zeta potential: −21.4 mV	Sustained-release up to 30 days; noncytotoxic; inhibit IL1β, TNFα and ROS	None	None	Gómez-Gaete et al. (2017)
CS	PLGA MS	Particle sizes: 75–500 μm; EE%: 70–80%	Microsphere size-dependent release; multiple burst release noncytotoxic	None	None	Jiang et al. (2011)
	Au-NPs	Average particle size: 13nm	Promote cell proliferation; Increase GAG and collagen production	None	None	Dwivedi et al. (2015)

Abbreviations: Dcn: Diacerein; SLN: solid lipid nanoparticles; GNPs: gold nanoparticles; Glc: gelucire; TPGS: d-α-tocopheryl polyethylene glycol 1000 succinate; HPMC: hydroxypropylmethyl cellulose; ROS: reactive oxygen species; EA: edge activator; CS: chondroitin sulfate; Au: gold.

Chondroitin sulfate (CS) is a sulfated GAG and an important component of the cartilage extracellular matrix (Alessio et al., 2021). It is widely used in the adjuvant therapy of OA due to its anti-inflammatory, anti-oxidative and anti-apoptotic effects (Henrotin et al., 2010). Studies showed that polymer-modified CS significantly increased the inter-tissue retention time. CS-encapsulated PLGA copolymers with different lactide and glycolide ratios showed different CS burst releases, and this may be potential for controlled drug release (Jiang et al., 2011). Priyanka Dwivedi (Dwivedi et al., 2015) et al combined gold NPs with CS to reinforce drug delivery. AuNps-CS was demonstrated to enhance chondrocyte proliferation and promote ECM production *in vitro*. Besides, a novel polymer agent formulated by CS-cysteine conjugate showed remarkable bioadhesive properties and low biotoxicity in rat primary chondrocytes (Suchaoin et al., 2016).

### 4.3. DDS targeting GAG loss and ROS of chondrocyte

Glucocorticoids have obvious anti-inflammatory effects, but long-term repeated use of large doses can lead to a variety of local or systemic side effects. Dexamethasone (Dex) is the most commonly used glucocorticoid for OA treatment. In order to improve the efficiency of drug use and reduce side effects, a variety of DDS has been developed. Bajpayee (Bajpayee et al., 2016; 2017) et al conjugated avidin nano-carriers with Dex by two linkers (ester and hydrazone). Ester linker had faster drug release than hydrazone linker and avidin-Dex rescued IL-1-induced GAG loss with does dependence. Subsequent animal experiments confirmed that the avidin-Dex could penetrate cartilage and retain for 3 weeks, improve morphology and inhibit the formation of osteophytes, but increased Dex load was needed to further reduce the loss of GAG. Dex-carbon nanotubes were developed to restrain TNF-α induced inflammation in synovial fibroblasts (Lee et al., 2017). Nanotubes showed higher Dex uptake by caveolin-dependent endocytosis and efficient intracellular release to inhibited ROS production by targeting mitochondria.

Stefano Perni (Perni & Prokopovich, 2020) et al greatly increased the drug uptake of Dex-poly-beta-amino-esters (PBAEs) by continuously optimizing the polymer structure. Dex-PBAEs inhibited GAG loss induced by IL-1α in cartilage explants cultured and improved the chondrocyte activity. Dex-loaded PLGA MP showed significant sustained release, pro-anabolic and anti-inflammatory factor effects *in vitro* and *in vivo* (Stefani et al., 2020). Tengfei He (He et al., 2020) et al developed a novel multi-arm avidin NPs, which greatly increased the drug load with crosslinkers. These multi-arm avidin NPs showed remarkable control of drug release and cartilaginous permeability. *In vitro* tests indicated that it inhibited the generation of ROS, protected the activity of chondrocytes, and reduced the loss of GAG and collagen.

The glucocorticoid-loaded DDS shows great potential for OA intraarticular targeted treatment. At the same time, some studies confirmed that the biological effects of the NPs DDS are affected by the dynamic changes of the joint environment, such as the changes of proteins, hyaluronic acid and phospholipids in the synovial fluid (Magri et al., 2019).

### 4.4. Novel drug molecules targeting osteanagenesis

In recent years, some new drug molecules introduced to different DDS showed remarkable therapeutic effects of OA in experiments, which has laid a foundation for clinical application (Table 3). Human stromal cell-derived factor 1α (rhSDF-1α) is a significant chemokine facilitating stem cell migration and homing to injured tissue and promoting tissue repair (Hattori et al., 2001). rhSDF-1α-loaded fibrin/HA hydrogel was used to filled chondral defects and it recruited chondrogenic progenitor cells to chondral defects, which improved the morphology, proteoglycan density and cartilage ultrastructure (Yu et al., 2015).

**Table 3.** Novel drug molecules and delivery system in OA treatment.

Drugs	Delivery system	Physical properties	*In vitro* findings	Rat model	*In vivo* performance	Refers
rhSDF-1α	Fibrin/hyaluronic acid hydrogel	None	Sustained-release up to 14 days; biocompatible; recruit chondrogenic progenitor cell; regeneration of cartilage tissue	None	None	Yu et al. (2015)
IGF-1	PAMAM dendrimers	Generation 6: 58 kDa	No histotoxicity	ACLT + MMx surgery	Penetrate cartilage; Sustained-release 30.4 days; rescue cartilage degeneration; reduces osteophyte formation	Geiger et al. (2018)
BMP2	GO flakes	Most abundant size: 1,598.5 nm	Sustained-release up to 40 days; inhibit inflammation; promote differentiation	ACLT surgery	Histological improvement; inhibit inflammation	Zhong et al. (2017)
TGF-β3	GO 3D nano-scaffold	Average size 10–40 μm; drug adsorb >99 %	Sustained-release up to 28 days; no cytotoxicity; promote chondrogenic differentiation	None	None	Zhou et al. (2019)
Glutathione	Chitosan-gelatin based hydrogel	Pore size: 1.667 μm	No cytotoxicity; sustained-release up to 48 h; rescue oxidative damage; down-regulate inflammation	None	None	Cheng et al. (2017)
KGN	PEG-PAMAM	Average size: 33.3– 36.4 nm; drug loading: 5.50 ± 0.23 (wt. %); Zeta potentials: +5 mV	Low cytotoxicity; promote chondrogenic differentiation and ECM production;	Healthy and 4% papain injection	Sustained-release up to 21 days	Hu et al. (2017)
	NPPs	Average size: 320 nm; drug loading: 31.5% (w/w)	Sustained release 62% drug in 3 months; bio-safe	DMM surgery	Histological improvement; cartilage protection; inhibit inflammation	Maudens et al. (2018)
	3D Tri-Copolymer Scaffolds	Mean pore size: 20 to 30 µm	Up-regulate Acan, Sox9, and Col2a1; Low cytotoxicity; improve chondrogenesis	None	None	Chen et al. (2021)
TPCA-1	Nanosomes	Average size: 50–200 nm	Low cytotoxicity; inhibit NO, LDH, PGE2, EPAS-1, MMP-13, ACAN and COL2A1	None	None	Bhatti et al. (2019)
Adenosine	PLA-b-PEG NPs	Diameter size: 129–144 nm	None	ACLT surgery	Inhibit NF-kb activation; chondroprotective; histological improvement	Liu et al. (2019)
HCQ	CMFn nanocages	Diameter:22 nm; loading ratio: 48%; embedding ratio: 5%	MMP-13/pH-responsive release; low cytotoxicity; increase Col2a1 and decrease inflammatory factors	None	None	Chen et al. (2019)

Abbreviations: rhSDF-1α): Human stromal cell-derived factor 1α; PAMAM: Amine terminal polyamidoamine; GO: Graphene oxide; KGN: Kartogenin; NPPs; nanocrystal–polymer particles; TPCA-1: [5-(p-Fluorophenyl)-2-ureido] thiophene-3-carboxamide; LDH: lactic dehydrogenase; PGE2: Prostaglandin E2; HCQ: hydroxychloroquine; CMFn: ferritin nanocages; DMM: Destabilization medial meniscus.

Anabolic growth factors are efficient for OA treatment by enhancing chondrocyte activity and promoting matrix production. An earlier study found that insulin-like growth factor 1 (IGF-1) fused to heparin-binding domain had a distinct prolongation of intraarticular retention and rescued cartilage degeneration in the rat OA model (Loffredo et al., 2014). Brett C Geiger (Geiger et al., 2018) et al loaded IGF-1 on positively charged PEGylated polyamidoamine (PAMAM) dendrimers and this dendrimer-IGF-1 presented prominent performance of drug absorption, cartilage penetration, drug retention and biocompatibility *in vitro*. The nanocarriers enhanced the treatment effects of alleviating cartilage degeneration and osteophyte formation *in vivo*. BMP2 is an important excitoanabolic factor in bone metabolism. BMP2 adsorbed onto graphene oxide (GO) flakes showed remarkable biocompatibility and sustained slow release *in vitro*. In the OA rat model, GO-adsorbed BMP2 had a better histological appearance after intra-articular intervention (Zhong et al., 2017). GO-loaded TGF-β3 3 D nano-scaffold is a new progress of cartilage engineering. Culture of hMSCs encapsulated in 3 D GO scaffold-adsorbed TGF-β3 hydrogel improved chondrogenesis and ECM production. This novel 3 D GO scaffold demonstrated excellent drug delivery, low cytotoxicity and sustaining drug activity (Zhou et al., 2019).

Yung-Hsin Cheng (Cheng et al., 2017) et al developed a glutathione-loaded chitosan hydrogel and used it in Cisd2 deficiency-induced rat chondrocytes injury. The hydrogel showed thermosensitively and sustained drug release in chondrocytes without obvious cytotoxicity. Glutathione rescued the inflammation, apoptosis and oxidative stress in Cisd2^–^/^–^ chondrocytes by restraining H_2_O_2_ activity.

Kartogenin (KGN) is found an important activator of the CBFβ-RUNX1 signaling pathway which promotes chondrogenesis and chondroprotection (Zhao et al., 2020). KGN was first conjugated to the head or end group of PEG-PAMAM to form KGN- PEG-PAMAM (KPP) or PEG-PAMAM-KGN (PPK) dendrimer. KPP showed the more prominent effect of CBFβ and chondrogenic markers activation. In vivo test showed prolonged retention of drug in a rat model (Hu et al., 2017). Pierre Maudens et al. (Maudens et al., 2018) introduced KGN nanocrystals acquiring by wet milling to PLA nanocrystal–polymer particles to form KGN-NPPs. The NPPs system presented commendable drug loading, sustained drug release and biocompatibility *in vitro* test. The KGN-loaded NPPs improved chondrohistology and osteophyte size *in vivo* test. In the past two years, tri-copolymer scaffolds structured by gelatin-chondroitin-hyaluronan and engineered exosomes were used to deliver KGN to chondrocytes and showed potential application future (Chen et al., 2021; Xu et al., 2021).

Nano-sized liposomes conjugated to MAbCII were synthesized to encapsulate TPCA-1, a selective inhibitor of NF-κB pathway, and showed distinguished improvement of inflammation, oxidative stress and cell apoptosis in TNF-α-treated chondrocytes (Bhatti et al., 2019). The advantage of liposomes is the sustained drug release properties but once the release is activated, it does not extend the clearance time. Xiuling Liu (Liu et al., 2019) et al conjugated adenosine to biodegradable PLA-b-PEG NPs in different binding sites. In vitro studies showed that adenosine-loaded NPs can significantly increase intracellular cAMP and inhibit a variety of inflammatory factors. Early injection of adenosine-loaded NPs into rat joints can effectively prevent traumatic OA.

Haimin Chen (Chen et al., 2019) et al optimized the MMP/pH-responsive DDS by synthesizing ferritin nanocages (CMFn) loaded with hydroxychloroquine (HCQ). The fluorescence intensity of CMFn can reflect the severity of OA and HCQ can be sustained released for 14 days in an acidic pH microenvironment. This dual sensitive DDS has great application prospects for precision OA diagnosis and treatment.

### 4.5. Chinese herb extracts and targeted DDS

Curcumin is extracted from the Chinese herb *Curcuma longa* which is commonly used in OA for inflammation and pain relief. Combined injection of curcumin and bone marrow mesenchymal stem cells (BMSCs) into the OA rat model can enhance the migration and proliferation of chondrocytes, improve the level of anabolic factors and promote chondrogenesis (Zhang et al., 2021). Regardless of oral, direct joint injection or transdermal administration, Curcumin shows obvious anti-OA effects and the potential mechanism is related to inhibition of oxidative stress, promotion of anabolism, and anti-inflammatory apoptosis (Nicoliche et al., 2020; Zhou et al., 2020). However, the traditional administration still has drawbacks such as multiple dosing and gastrointestinal side effects, although the study showed better tolerance of curcumin than that of Dcf (Shep et al., 2019). Some studies used gelatin/silk fibroin MPs and synthetic NPs loaded with curcumin and showed good drug penetration and sustained release (Zhang et al., 2016; Ratanavaraporn et al., 2017).

Qiumei Lan (Lan et al., 2020) et al developed another MMP/pH-responsive DDS loading psoralidin (PSO) which is extracted from *pPoralea corylifolia*. The MRC-PPL NPs are designed to target cartilage and respond to MMP-13. MRC-PPL@PSO showed remarkable anti-inflammatory and cartilage repair effects *in vitro* and *in vivo* test by regulating PI3K/AKT, MAPK and NF-κB signaling. Zhengxiao Ouyang et al. (Ouyang et al., 2019) introduced hesperetin (extracted from citrus fruit) to Gd2(CO3)3-based NPs with a cartilage-targeting peptide. The NPs displayed excellent biocompatibility and magnetic resonance suitability. Hesperetin showed remarkable chondrocytes protection by inhibiting the TLR-2/NF-κB/Akt pathway.

Salvianolic acid A (SAA) is extracted from *Salvia miltiorrhiza* Bunge and it can inhibit chondrocyte apoptosis and ECM degradation in the OA rat model by restraining NF-κB signaling and activating TIMP-1 and TIMP-2 to inhibit MMPs (Xu et al., 2017; Wu et al., 2020). Artesunate (ART) is derived from artemisinin which is an extract of Chinese herb used to treat malaria. ART can relieve OA rat inflammation and improve cartilage pathological by regulating AK/STAT signaling (Zhao et al., 2017). Ethanol extract of *Agkistrodon acutus* can alleviate the apoptosis of chondrocytes and correct the abnormal expression of MMPs and Col2in OA rats (Wang et al., 2019). These Chinese herb extracts demonstrate remarkable effects in OA, and they have tremendous potential to work with appropriate DDS in the clinical application (Table 4).

**Table 4.** Chinese herb extracts and gene delivery in OA treatment.

Drugs	Delivery system	Physical properties	*In vitro* findings	Rat model	*In vivo* performance	Refers
Curcumin	Gelatin/silk fibroin microspheres	EE%: 55–59%; Low degradation	Low degradation; SUSTAINED release over 14 days	50 µl of MIA (60 mg/ml)	Inhibit development of OA; Histological improvement	Ratanavaraporn et al. (2017)
PSO	MRC-PPL NPs	Average size: 121.5 ± 26.1 nm Zeta potential: −5.75 mV; Loading percentage: 16.9%	pH responsive release; No cytotoxicity with 15 μM PSO; Increase Col2a1 and decrease MMP-13 and TNF-α	Papain solution (8% W/V) and l-cysteine (0.03 mol/L)	Sustained-release up to 21 days; low organ uptake; cartilage protection; reduce osteophyte; histological improvement	Lan et al. (2020)
Hes	PDA-Gd2(CO3)3 NPs	Loading percentage: 9.32%; anchoring efficiency: 67.86 %	Nontoxic; cartilage affinity; attenuate apoptosis and inflammation; inhibit TLR-2 and NF-κB/Akt	ACLT surgery	MR suitability; low organ uptake; biocompatibility and low cytotoxicity; histological improvement	Ouyang et al. (2019)
mASOs	PEG-SWCNT-anti-GFP	An average value of hm: 3.1 ± 1.2 nm	None	DMM surgery	Inhibit development of OA; prolonged retention time; enter the ECM; deliver gene into chondrocytes	Sacchetti et al. (2014)
Ihh	Lipid NPs	EE%: above 95%; diameters: 67 ± 4.3 nm;	Excellent penetration; low cytotoxicity;	Healthy and ACLT surgery	Inhibit Ihh expression; increase anabolic factors; attenuate OA pathogenesis	Thompson et al. (2015)
antimiR-221	fibrin/HA hydrogel	None	Sustained release over 14 days; miR-221 knockdown in 7 days; increase anabolic factors	Osteochondral biopsy	Enhanced cartilage repair	Lolli et al. (2019)
NF-κB p65 siRNA	Self-assembling peptidic NPs	Average size: 55 ± 18 nm; zeta potential: 12 ± 0.7 mV	Suppresses p65; preserve chondrocyte viability; maintains cartilage homeostasis	None	None	Yan et al. (2019)

Abbreviations: PSO: psoralidin; MR-PPL: MR-Cy5.5-BHQ-3-PPL; Gd2(CO3)3: PDA: polydopamine; Hes: Hesperetin; SAA: Salvianolic acid A; SWCNTs: single-walled carbon nanotubes; anti-GFP: anti-green fluorescent protein; Ihh: Indian Hedgehog.

### 4.6. Gene delivery system

Transfering exogenous nucleic acids to intracellular compartments is an effective method for OA treatment. Gene therapy includes DNA, RNA and no-coding RNA that are specific to different diseases and tissues. Nanomaterial non-viral vectors can avoid the immunogenicity, oncogenic effects and other lethality of traditional viral vectors and become a promising gene therapy DDS (Table 4). Cristiano Sacchetti et al. (Sacchetti et al., 2014) used single-walled carbon nanotubes (SWCNTs) to load morpholino antisense oligonucleotides (mASOs) modified by anti-green fluorescent protein (GFP). Intra-articular injection of PEG-SWCNT-anti-GFP mASOs can effectively penetrate the ECM, deliver drugs to the chondrocytes with a prolonged retention time and inhibit GFP expression.

Indian Hedgehog (Ihh) is a non-collagen related marker which is closely related to chondrocyte injury and the development of OA (Zhang et al., 2012; Thompson et al., 2015). Lipid NPs were used to load Ihh siRNA into chondrocytes with 100% transfection efficiency. Lipid NPs-Ihh siRNA was found to accumulate in the ECM instead of the synovium. In addition, it showed remarkable excito-anabolic and anti-catabolism effects and effectively alleviated cartilage degeneration in the OA rat model (Wang et al., 2018). The fibrin/HA hydrogel and self-assembling peptidic NPs were also used for carrying antimiR-221 and NF-κB p65 siRNA, respectively. These DDS showed excellent targeting delivery, gene silencing and OA therapeutic effects (Lolli et al., 2019; Yan et al., 2019).

### 4.7. Multidrug delivery system

Some formulations use targeted delivery of combination drugs, which makes the therapeutic effect more comprehensive. Mamta Bishnoi et al. (Bishnoi et al., 2014) conjugated aceclofenac-loaded CS to SLN and the SLNs showed sustained drug release over 24 hours *in vitro* test. When administered subcutaneously, SLNs presented high concentrations in the joints but no significant accumulation in vital organs and reduced edema induced by MIA. KGN and Dcf were conjugated to thermoresponsive NPs outside and inside, respectively (Kang et al., 2016). The therapeutic effects of both drugs were fully demonstrated in the formula, and the drugs could be released with precise control in cold temperatures. Similarly, indomethalin and glucosamine were jointly loaded on PLGA nano-micelles and observably improved inflammatory response and histopathology in the OA rat model (Kamel et al., 2016).

Xu Chen (Chen et al., 2019) et al developed a photothermal-triggered nanogenerator which loaded NO and Notch1-siRNA on PLGA-PEG NPs. With the synergistic effect of phototherapy, this formula achieved NO anti-inflammatory effect, Notch1 gene silencing, and alleviation of cartilage erosion. The combination of multidrug delivery and physical factor therapy is a promising approach for OA treatment.

## 5. Conclusions and future prospects

The constant development of DDS displays a great avenue for targeted therapy of OA. Traditional drugs fully release the therapeutic potential and greatly reduce the drawbacks and side effects accompanied by systematic administration. Many emergent drug molecules present great therapeutic potential *in vitro* and *in vivo* studies, whether in inflammation suppression, chondrocyte protection, extracellular matrix generation, or the relief of corresponding symptoms. The future application of these drugs still needs more explorations to confirm and optimize the property, and help reveal the internal mechanism of the occurrence and development of OA.

Nano-DDS is an important optimization of OA topical administration, which realizes biological functions such as drug penetration, long-term retention and sustained release. By modulating DDS structure, the encapsulation efficiency of the drug is greatly improved, which avoid large dose or frequent administration. The penetration of chondrocyte ECM in DDS has made some progress, but it is still one of the difficult problems to be solved in the later period. Multi-structure integration of NPs can be loaded with different types of drugs, which can help enrich therapeutic strategies according to the specific condition of the disease. Responsive nano DDS opens a new horizon for the precise treatment of OA. Drug release controlled by joint microenvironment changes can help better grasp the disease progress, while the physical factor response system takes full advantage of physiotherapy on the basis of precision treatment.

Nanotechnology has revealed the great plasticity of new materials in the medical field and these findings will catalyze new breakthroughs of DDS. In future studies, it is necessary to more clearly reveal the pathogenesis of OA and find more drugs with definite efficacy to enrich treatment strategies. At present, many studies get remarkable results *in vitro* or ex vivo, but there is still a long way to go before clinical application. Each drug delivery strategy needs to be reevaluated for safety, consistency, and clinic efficiency over long-term clinical studies. The drug load is an important translation challenge due to the differences in drug requirements for human OA and animal studies. Biocompatibility, low biotoxicity, and biodegradability remain the primary concerns for the development of DDS. The nanomaterials need to be further optimized to achieve good human adaptability, and the pharmacokinetics of the drug loading system need to be clarified. In addition, the structure-function relationship between nanomaterials and different stages of OA (changes in synovial fluid, cartilage, subchondral bone, muscle, ligament and other tissues as well as the intraarticular environment) is also important challenge for clinical conversion.
